# Acute Ethanol Intake Induces NAD(P)H Oxidase Activation and Rhoa Translocation in Resistance Arteries

**DOI:** 10.5935/abc.20160147

**Published:** 2016-11

**Authors:** Janaina A. Simplicio, Ulisses Vilela Hipólito, Gabriel Tavares do Vale, Glaucia Elena Callera, Camila André Pereira, Rhian M Touyz, Rita de Cássia Tostes, Carlos R. Tirapelli

**Affiliations:** 1 Departamento de Enfermagem Psiquiátrica e Ciências Humanas - Laboratório de Farmacologia - Escola de Enfermagem de Ribeirão Preto - Universidade de São Paulo (USP); SP, Brazil; 2 Programa de Pós-Graduação em Farmacologia - Faculdade de Medicina de Ribeirão Preto - Universidade de São Paulo (USP), SP, Brazil; 3 University of Ottawa, Canada

**Keywords:** Ethanol, NADPH Oxidase, Rats, Oxidative Stress, Ascorbic Acid, rhoAGTP-Binding Protein

## Abstract

**Background:**

The mechanism underlying the vascular dysfunction induced by ethanol is not totally understood. Identification of biochemical/molecular mechanisms that could explain such effects is warranted.

**Objective:**

To investigate whether acute ethanol intake activates the vascular RhoA/Rho kinase pathway in resistance arteries and the role of NAD(P)H oxidase-derived reactive oxygen species (ROS) on such response. We also evaluated the requirement of p47phox translocation for ethanol-induced NAD(P)H oxidase activation.

**Methods:**

Male Wistar rats were orally treated with ethanol (1g/kg, p.o. gavage) or water (control). Some rats were treated with vitamin C (250 mg/kg, p.o. gavage, 5 days) before administration of water or ethanol. The mesenteric arterial bed (MAB) was collected 30 min after ethanol administration.

**Results:**

Vitamin C prevented ethanol-induced increase in superoxide anion (O_2_^-^) generation and lipoperoxidation in the MAB. Catalase and superoxide dismutase activities and the reduced glutathione, nitrate and hydrogen peroxide (H_2_O_2_) levels were not affected by ethanol. Vitamin C and 4-methylpyrazole prevented the increase on O_2_^-^ generation induced by ethanol in cultured MAB vascular smooth muscle cells. Ethanol had no effect on phosphorylation levels of protein kinase B (Akt) and eNOS (Ser^1177^ or Thr^495^ residues) or MAB vascular reactivity. Vitamin C prevented ethanol-induced increase in the membrane: cytosol fraction ratio of p47phox and RhoA expression in the rat MAB.

**Conclusion:**

Acute ethanol intake induces activation of the RhoA/Rho kinase pathway by a mechanism that involves ROS generation. In resistance arteries, ethanol activates NAD(P)H oxidase by inducing p47phox translocation by a redox-sensitive mechanism.

## Introduction

Excessive consumption of ethanol, often referred in the literature as "binge drinking", is considered a risk for the cardiovascular system. Binge drinking is associated with an increased risk of cardiovascular events, such as atherosclerosis, stroke and myocardial infarction.^1-3^ The exact mechanism underlying the cardiovascular dysfunction induced by binge drinking is not totally understood; but changes on vascular function may play a key role.^4-6^

One important mechanism by which ethanol leads to vascular damage is by increasing the generation of reactive oxygen species (ROS). Ethanol-mediated generation of superoxide anion (O_2_^-^) and hydrogen peroxide (H_2_O_2_) is associated with vascular dysfunction.^7,8^ The enzyme nicotinamide adenine dinucleotide phosphate [NAD(P)H] oxidase is the main source of ROS in endothelial and vascular smooth muscle cells (VSMC),^9^ and a an essential factor in ethanol-induced vascular dysfunction.^10^ The activation of NAD(P)H oxidase is a complex process , but translocation of the cytosolic subunit p47phox to the membrane, with further association with the cytochrome b_558_, is a decisive step for the activation of the enzyme .^11^ Recent findings from our laboratory have shown that acute ethanol intake increases the generation of NAD(P)H oxidase-derived ROS in resistance arteries.^12^

ROS activate downstream signaling targets, including mitogen-activated protein kinases (MAPK) and RhoA/Rho kinase, which are considered important mediators of vascular dysfunction. Activation of these redox-sensitive pathways regulates vascular cell growth, inflammation and contraction.^13^ Importantly, acute ethanol intake induces NAD(P)H oxidase activation and MAPK phosphorylation in resistance arteries.^12^ Additionally, the rapid inactivation of nitric oxide (NO)^14^ induced by O_2_^-^, and changes in NO synthesis and/or bioavailability have been implicated in the development of clinically significant vascular events.

In the present study, we sought to investigate whether acute ethanol intake activates the vascular RhoA/Rho kinase pathway in resistance arteries and the role of NAD(P)H oxidase-derived ROS on such response. Additionally, we evaluated the requirement of p47phox translocation for the activation of NAD(P)H oxidase induced by ethanol and the effect of ethanol-induced ROS on NO production. Ascorbic acid (vitamin C) was chosen as the antioxidant since it has been described to reduce oxidative stress in the vasculature.^15,16^

## Methods

### Acute ethanol administration

A dose of 1g/kg of ethanol (10 mL/kg of 13% ethanol diluted in water) was administered by gavage to 20 male Wistar rats (200-250 g) fasted for 12 h.^12,16^ Blood ethanol levels using this model of ethanol administration are within the range of 20-24 mmol/L.^12,16,17^ Rats from the control group (n=20) received water (gavage). Some rats were treated with vitamin C at a dose of 250 mg/kg (gavage) for 5 days,^16,18,19^ before administration of water (n=18) or ethanol (n=19). The sample size was based on previous studies.^12,16,17^ The mesenteric arterial bed (MAB) was isolated from the rats 30 min after ethanol administration.^17^ All experiments were in accordance with the principles and guidelines of the animal ethics committee of the University of São Paulo (#10.1.235.53.0).

### Detection of O_2_^-^ in the rat MAB

Lucigenin-derived chemiluminescence assay was performed to measure O_2_^-^ production in the rat MAB as previously described.^12^ Luminescence was measured in a luminometer (Orion II Luminometer, Berthold detection systems, Pforzheim, Germany) and the results are expressed as relative light units per mg of protein. Protein concentrations in the samples were measured using the method of Lowry (Bio-Rad Laboratories, Hercules, CA, USA).

### Detection of H_2_O_2_ in the rat MAB

In order to measure H_2_O_2_ concentration in the rat MAB, Amplex red^®^ (#A22188, Invitrogen, Carlsbad, CA, USA) was used as previously described.^20^ Results are expressed as nmol H_2_O_2_/mg protein

### Detection of basal nitrate in the rat MAB

Baseline nitrate concentrations of supernatants from MAB homogenates was evaluated using a Sievers Chemiluminescence Analyzer (Nitric Oxide Analyser, NOA^TM^ 280, Sievers Instruments, CO, USA) as previously described.^12^ Results are expressed as *µ*mol/L per mg of protein.

### Evaluation of superoxide dismutase (SOD) and catalase (CAT) activities in the rat MAB

The activity of SOD in the rat MAB was evaluated using a commercially available kit (#19160, Sigma-Aldrich, St. Louis, MO, USA). SOD activity is expressed as inhibition rate % per mg of protein. The activity of CAT was determined as previously described.^12^ One CAT unit (U) was defined as the amount of enzyme required to decompose 1 *µ*mol of H_2_O_2_/min.

### Evaluation of reduced glutathione (GSH) concentration in the rat MAB

The concentration of GSH in the rat MAB was determined as previously described.^12^ Results are expressed as *µ*g GSH per mg of protein.

### Evaluation of thiobarbituric acid reactive substances (TBARS) in the rat MAB

The concentration of TBARS in the rat MAB was determined using a commercially available kit (#10009055, Cayman Chemical, Ann Arbor, MI, USA). Results are expressed as nmol per mg of protein.

### Immunoblotting

The rat MAB was homogenized in a lysis buffer composed of 50 mmol/L Tris-HCl (pH 7.4), NP-40 (1%), sodium deoxycholate (0.5%) and SDS (0.1%). Samples were centrifuged at 5,000 × g for 10 min (4ºC). Forty micrograms of protein were separated by electrophoresis on a 10% polyacrylamide gel, and transferred onto a nitrocellulose membrane. Skimmed milk 5% diluted in Tris-buffered saline solution with Tween 20 was used to block nonspecific binding sites (1 h at 24ºC). Membranes were then incubated overnight at 4ºC with the following primary antibodies: p-eNOS (Ser^1177^) (diluted 1:1000, 9571, Cell Signaling, Danvers, MA, USA), p-eNOS (Thr^495^) (diluted 1:1000, 9574, Cell Signaling), total eNOS (diluted 1:1000, 9572, Cell Signaling), P-protein kinase B (P-Akt) (Ser^473^) (diluted 1:1000, 4058, Cell Signaling) and total Akt (diluted 1:1000, 9272, Cell Signaling). Membranes were then incubated with secondary antibodies for 90 min at room temperature. Signals were revealed with chemiluminescence and quantified densitometrically. The results are expressed as the non-phospho/total proteins ratio.

### Cytosol/membrane fractionation

The rat MAB was homogenized in lysis buffer containing 50 mmol/L Tris-HCl (pH 7.4), 2.5 mmol/L EDTA, 5 mmol/L EGTA, and protease inhibitor. Homogenates were centrifuged at 100,000 × g for 1 h, at 4ºC. The supernatant (cytosolic fraction) was collected. The pellet, containing the particulate fraction, was resuspended in lysis buffer containing 1% Triton X-100 and centrifuged at 10,000 × g for 10 min at 4 °C. Membranes were then incubated with specific antibodies for RhoA (diluted 1:1000, sc-418, Santa Cruz Biotechnology, Dallas, TX, USA) or p47phox (diluted 1:500, Santa Cruz Biotechnology) as previously published.^21^

### Vascular reactivity experiments

Male Wistar rats were anesthetized with urethane (1.25 g/kg, i.p., Sigma-Aldrich, St. Louis, MO, USA) and killed by aortic exsanguination. Segments of third-branch mesenteric arteries, measuring about 2 mm in length, were mounted in a small vessel myograph (Danish Myo Tech, Model 620M, A/S, Århus, Denmark) as previously described.^22^ Arteries were maintained in Krebs Henseleit solution [(in mmol/L) 130 NaCl, 4.7 KCl, 1.18 KH_2_PO_4_, 1.17 MgSO_4_, 14.9 NaHCO_3_, 5.5 glucose, 0.03 EDTA, 1.6 CaCl_2_)], at a constant temperature of 37°C, pH 7.4 and gassed with a mixture of 95% O_2_ and 5% CO_2_. Concentration-response curves to phenylephrine (0.1 nmol/L-100 *µ*mol/L) were performed in endothelium-intact and endothelium-denuded arteries. The curves for acetylcholine (0.1 nmol/L-10 *µ*mol/L) were performed in endothelium-intact arteries, pre-contracted with phenylephrine (1 *µ*mol/L). Concentration-response curves were fitted using a nonlinear curve fitting program (Graph Pad Prism 3.0; GraphPad Software Inc., San Diego, CA). Two pharmacological parameters were analyzed: pD_2_ (negative logarithm of the molar concentration of the drug producing 50% of the maximum response) and E_max_ (maximum effect elicited by the agonist).

### Cell culture and stimulation

VSMC derived from the MAB of male Wistar-Kyoto rats were isolated and characterized as previously described.^23^ Cells at passages 4 to 8 isolated from at least 5 different primary cell cultures were used. VSMC were stimulated with ethanol (50 mmol/L, 5 min) in the absence or in the presence of apocynin, a NAD(P)H oxidase inhibitor (10 *µ*mol/L, 30 min), tiron (O_2_^-^ scavenger, 10 *µ*mol/L, 30 min), 4-methylpyrazole (4-MP,10 *µ*mol/L, 30 min), a selective alcohol dehydrogenase (ADH) inhibitor, or vitamin C (100 *µ*mol/L, 24 h). The concentration and period of exposition to ethanol and antioxidants were based on previous studies.^12,24^ Superoxide anion production was measured by lucigenin-enhanced chemiluminescence as described above, and is expressed as percentage increase from baseline values.

### Statistical analysis

Data are presented as mean ± standard error of the mean (SEM). Groups were compared using one-way analysis of variance (ANOVA) followed by Bonferroni's multiple comparison test. Data followed a normal distribution. Results of statistical tests with p<0.05 were considered as significant. Statistical analysis was carried out using the program Graph Pad Prism 3.0 (GraphPad Software Inc., San Diego, CA, USA).

## Results

### Blood ethanol levels

As we had previously reported,^12,16,17^ in this model of ethanol administration, blood ethanol levels vary from 20-24 mmol/L, which are within the range found in the bloodstream of humans after a binge drinking episode.^25^

### Effect of acute ethanol intake on O_2_^-^, H_2_O_2_, TBARS, nitrate and GSH levels and SOD and CAT activities in the rat MAB

**Figure 1:**
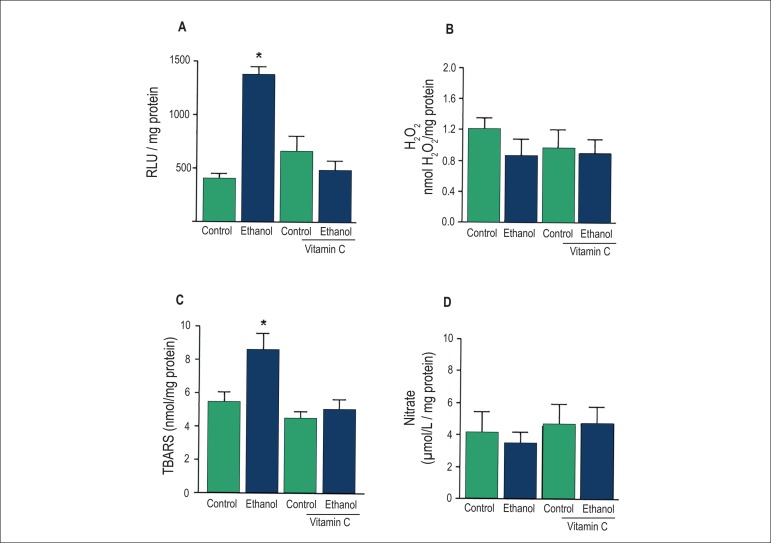
Effects of acute ethanol intake on O_2_^-^, H_2_O_2_ and nitrate levels in the rat mesenteric arterial bed . Vascular levels of O_2_^-^ (A) and nitrate (D) were determined by chemiluminescence. Vascular H_2_ O _2_ levels were measured fluorometrically (B). thiobarbituric acid reactive substances (TBARS) concentration was determined colorimetrically (C). Results are presented as means ± SEM of 6-9 experiments. *Compared to control, control plus vitamin C and ethanol plus vitamin C (p<0.05, ANOVA).

**Figure 2:**
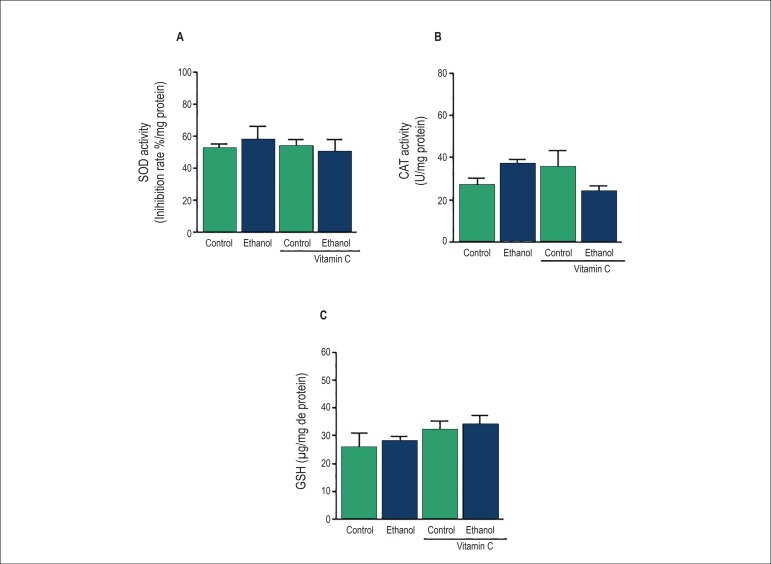
Effects of acute ethanol intake on superoxide dismutase (SOD) and catalase (CAT) activities and reduced glutathione (GSH) levels in the rat mesenteric arterial bed. SOD and CAT activities (A and B) and GSH levels (C) were determinate colorimetrically. Results are presented as means ± SEM of 6-8 experiments.

The effect of acute ethanol intake on ROS generation and lipid peroxidation in the rat MAB was evaluated by assessing the generation of O_2_^-^ and H_2_O_2_ and the concentration of TBARS. Lucigenin-derived luminescence was significantly higher in the MAB from ethanol-treated rats, and such response was prevented by treatment with vitamin C (Figure 1A). No changes in H_2_O_2_ levels were observed after treatment with ethanol (Figure 1B). Vitamin C prevented the increase on TBARS concentration induced by acute ethanol intake (Figure 1C). Since increased oxidative stress is associated with reduced NO bioavailability, we evaluated the effect of acute ethanol intake on nitrate concentration in the rat MAB. Ethanol treatment did not alter the baseline levels of nitrate in the rat MAB (Figure 1D). In order to evaluate the effect of ethanol on the vascular antioxidant status, SOD and CAT activities as well as GSH concentrations were determined in the rat MAB. Our results showed that treatment with ethanol had no effect on these parameters (Figure 2).

### Effect of ethanol on O_2_^-^ generation in cultured MAB VSMC

**Figure 3:**
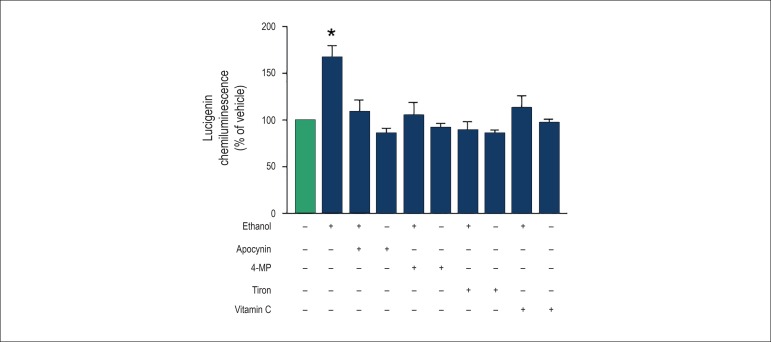
Effect of tiron, apocynin, 4-MP and vitamin C on ethanol-induced O_2_^-^ generation in cultured VSMC of the rat mesenteric arterial bed . Cells were stimulated with ethanol (50 mmol/L, 5 min) in the absence or after incubation with tiron (10 µmol/L, 30 min), apocynin (10 µmol/L, 30 min), 4-methylpyrazole (4-MP) (10 µmol/L, 30 min) or vitamin C (100 µmol/L, 24 h). The bars represent the mean ± SEM of 7-11 experiments. * Compared to vehicle (p<0.05, ANOVA).

The antioxidant effect of vitamin C was tested in cultured MAB VSMC. Vitamin C prevented the increase on O_2_^-^ generation induced by ethanol (50 mmol/L, 5 min) in cultured VSMC. To test the role of an ethanol metabolite on ethanol-induced O_2_^-^ generation, the cells were incubated with 4-MP, an ADH inhibitor. 4-MP prevented ethanol-induced O_2_^-^ generation in cultured VSMC. Apocynin and tiron also prevented the generation of O_2_^-^ induced by ethanol (Figure 3).

### Evaluation of Akt and eNOS phosphorylation in the rat MAB

**Figure 4:**
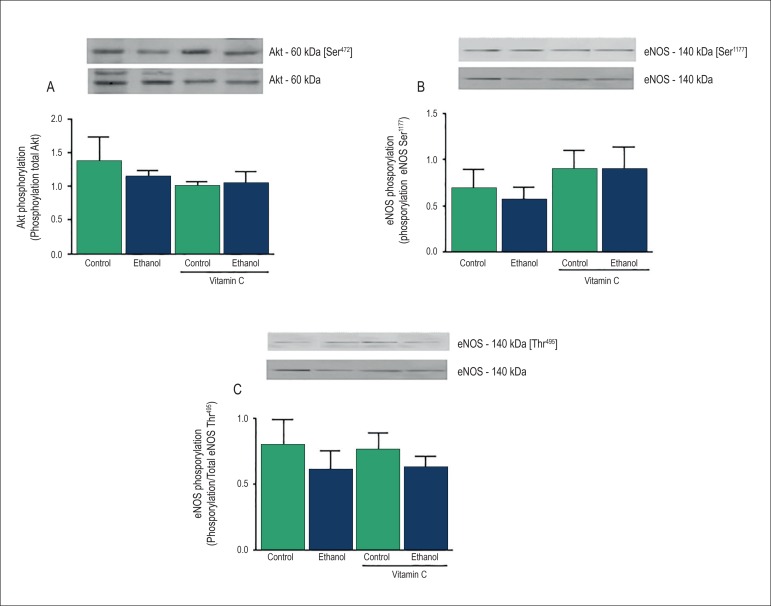
Effects of acute ethanol intake on protein kinase B (Akt) and endothelial nitric oxide synthase (eNOS) phosphorylation in the rat mesenteric arterial bed . Top panels: representative immunoblots for Akt and eNOS protein phosphorylation and expression. Bottom panels: corresponding bar graphs showing densitometric data for phosphorylation of Akt at Ser^473^ residue (A), eNOS at Ser^1177^ residue (B) and eNOS at Thr^495^ residue (C). Results are presented as mean ± SEM of 4-6 experiments.

Our findings showed no alteration in the phosphorylation of Akt at Ser^473^ residue or eNOS at Ser^1177^ and Thr^495^ residues after acute ethanol intake or treatment with vitamin C (Figure 4).

### Evaluation of p47phox and RhoA translocation in the rat MAB

**Figure 5:**
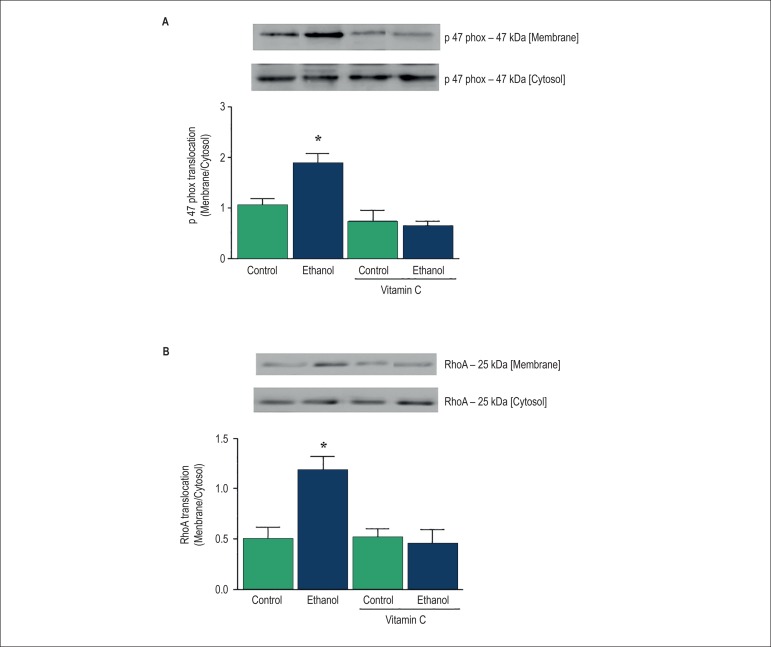
Effects of acute ethanol intake on p47phox and RhoA translocation in the rat mesenteric arterial bed (MAB). Bar graph represents translocation of p47phox and RhoA as a ratio of membrane/cytosol expression (A and B) in the rat MAB. Top panels: representative immunoblots. Results are presented as mean ± SEM of 5-7 experiments. *Compared with control, control plus vitamin C and ethanol plus vitamin C (p<0.05, ANOVA).

Since NAD(P)H oxidase is the major source of ROS in the vasculature, and NAD(P)H-derived ROS induce activation of the RhoA/Rho kinase signaling pathway, we evaluated the effect of ethanol on p47phox and RhoA translocation. MAB from ethanol-treated rats displayed a significant increase in the membrane/cytosol fraction ratio of p47phox (Figure 5A) and RhoA expression (Figure 5B), indicating the translocation of the proteins. Treatment with vitamin C prevented the increase in p47phox and RhoA translocation induced by ethanol.

### Experiments on vascular reactivity of mesenteric artery

**Figure 6:**
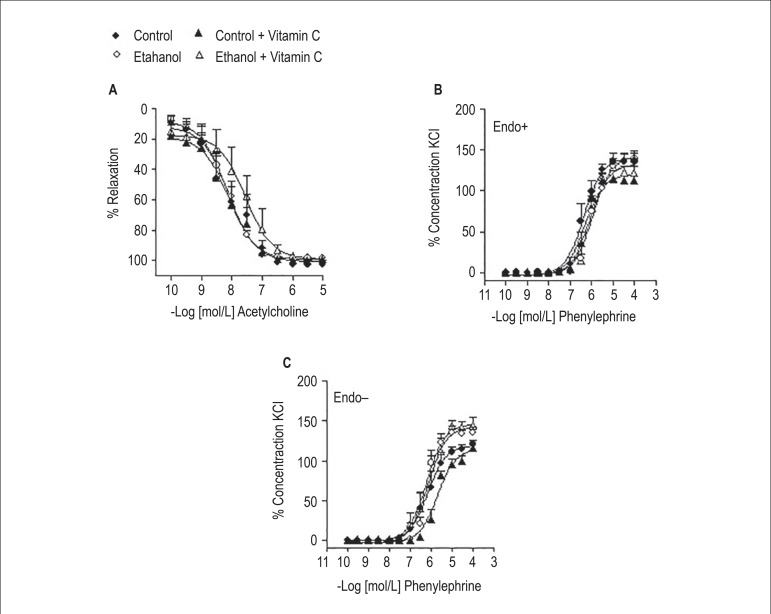
Effects of acute ethanol intake on vascular reactivity to acetylcholine and phenylephrine. Concentration-response curves for acetylcholine were performed in endothelium-intact third branch mesenteric arteries (A). Concentration-response curves for phenylephrine were obtained in endothelium-intact (Endo+, B) or endothelium-denuded (Endo-, C) arteries. Results are presented as means ± SEM of 4 to 6 experiments.

Ethanol treatment did not affect acetylcholine-induced relaxation (E_max_: 98.3 ± 1.5%; pD_2_: 8.2 ± 0.2, n=6), when compared to control (E_max_: 100.3 ± 2.3%; pD_2_: 8.1 ± 0.4, n=6), control plus vitamin C (E_max_: 98.6 ± 1.8%; pD_2_: 8.1 ± 0.2, n=4) and ethanol plus vitamin C (E_max_: 99.8 ± 0.4%; pD_2_: 7.8 ± 0.3, n=5) groups (Figure 6) . In endothelium-intact arteries, acute ethanol intake had no effect on contraction (% KCl 120 mmol/L) induced by phenylephrine (E_max_: 138.8 ± 10.4%; pD_2_: 5.9 ± 0.3, n=5), when compared to control (E_max_: 136.3 ± 10.3%; pD_2_: 6.3 ± 0.2, n=4), control plus vitamin C (E_max_: 112.9 ± 2.4%; pD_2_: 6.3 ± 0.1, n=4) and ethanol plus vitamin C (E_max_: 122.3 ± 7.7%; pD_2_: 6.1 ± 0.1, n=6) groups. Similarly, ethanol treatment did not alter the contraction induced by phenylephrine in endothelium-denuded arteries (E_max_: 135.1 ± 7.2%; pD_2_: 6.1 ± 0.1, n=6), when compared to control (E_max_: 120.7 ± 5.5%; pD_2_: 6.1 ± 0.3, n=5), control plus vitamin C (E_max_: 116.3 ± 3.5%; pD_2_: 5.7 ± 0.1, n=4) and ethanol plus vitamin C (E_max_: 144.3 ± 10.4%; pD_2_: 6.1 ± 0.3, n=5) groups.

## Discussion

The present findings show that acute ethanol intake induces RhoA translocation and NAD(P)H oxidase activation by promoting p47phox translocation in resistance arteries. Despite increasing ROS generation, acute ethanol intake does not affect NO synthesis or bioavailability in resistance arteries. The relevance of our findings is strengthened by previous studies of our group that showed that using this same model of ethanol administration, plasma ethanol levels are within the range of 20-24 mmol/L^12,16,17^ which corresponds to that found in humans after a "binge drinking" session ,^25^ and in rats 30 min after oral administration of ethanol.^26^

Our findings demonstrated that ethanol increased O_2_^-^ generation in the rat MAB, which is in accordance with previous results from our laboratory.^12^ Additionally, increased lipid peroxidation was observed in the rat MAB after ethanol intake. The lucigenin-derived chemiluminescence assay is based on the enzymatic action of the enzyme NAD(P)H oxidase.^27^ In this sense, the increase in chemiluminescence here described suggests that the enzyme NAD(P)H oxidase is an important source of acute ethanol-induced O_2_^-^ generation in resistance arteries. This idea is supported by the fact that apocynin inhibited ethanol-induced O_2_^-^ generation in cultured VSMC. Vitamin C is an effective scavenger of O_2_^-^^28^ and in our model it decreased ethanol-induced O_2_^-^ generation and lipid peroxidation in resistance arteries. The antioxidant property of vitamin C is also associated with decreased activation of NAD(P)H oxidase.^15^ In cultured VSMC, vitamin C prevented ethanol-induced O_2_^-^ generation, suggesting that the inhibition of NAD(P)H oxidase by vitamin C may also be implicated in diminishing ethanol-induced vascular O_2_^-^ generation.

ADH is an ethanol-metabolizing enzyme that is functionally active in the vasculature.^29^ We have previously described that ethanol metabolites are involved in the vascular effects elicited by ethanol.^30^ In order to determine a possible role for ethanol metabolites on O_2_^-^ generation induced by ethanol in resistance arteries, we evaluated the effect of 4-MP on this process. Inhibition of ethanol metabolism mediated by ADH led to a decrease in ethanol-induced O_2_^-^ generation in cultured VSMC. Thus, we present evidence that an ethanol metabolite, possibly acetaldehyde, is responsible for ROS generation in resistance arteries.

NAD(P)H oxidase is the major source of ROS in the vasculature. The prototypical phagocytic Nox comprises five subunits: three cytosolic regulatory subunits, p40phox, p47phox and p67phox and two membrane-associated components named gp91phox (Nox2) and p22phox. The enzymatic complex is dissociated in resting cells but it is rapidly activated upon cellular stimulation. Phosphorylation of p47phox at a serine residue initiates the activation of the enzyme, triggering the formation of a complex composed by the cytosolic subunits. This response is followed by translocation of the cytosolic complex to the membrane and association with gp91phox and p22phox subunits (cytochrome b_558_). NAD(P)H oxidase components are expressed in endothelial and VSMC, and translocation of the p47phox subunit has been shown to be essential for ROS production in these cells.^31^ In our study, the translocation of p47phox was increased in MAB from ethanol-treated rats, and this effect was inhibited by vitamin C, suggesting that oxidative stress is involved in ethanol-induced p47phox translocation and NAD(P)H oxidase activation. Moreover, this observation strengthens our initial idea that NAD(P)H oxidase is implicated in ethanol-induced O_2_^-^ generation in the rat MAB. The inhibitory action of vitamin C on p47phox translocation has been previously described.^32^ Mechanisms whereby vitamin C inhibits the translocation of p47phox are ill defined, but may involve the inhibition of NAD(P)H oxidase activators. A large number of proteins are involved in NAD(P)H oxidase assembly. These include Rac GTPases, protein kinase C (PKC) and c-Src.^11^ Papparella et al.^32^ showed that vitamin C inhibited PKC activation with subsequent reduction in p47phox translocation and ROS generation. The current study did not address the exact mechanism whereby ethanol modulates NAD(P)H oxidase activity. Different stimuli, such as endothelin-1, angiotensin II, catecholamines, thrombin and growth factors (eg.: epidermal and growth factor and platelet-derived growth factors ) acutely activate NAD(P)H oxidase in the vasculature.^33^ We have previously shown that losartan, an antagonist of AT_1_ receptors, did not prevent ethanol-induced ROS generation in the rat MAB,^12^ ruling out a role for angiotensin II in ethanol-induced NAD(P)H activation here described. Future studies designed to address how acute ethanol intake induces p47phox translocation and ROS generation in resistance arteries are of interest.

Superoxide anion is a highly unstable molecule that is reduced by SOD to H_2_O_2._^34^ In our study, the antioxidant action of vitamin C was not related to an increase in SOD activation, since no difference on SOD activity was observed in the MAB after treatment with the vitamin . In addition , since ethanol treatment did not alter SOD activity , the ethanol-induced increase in O_2_^-^ levels seems not to be related to decreased dismutation of O_2_^-^ by SOD. Although both O_2_^-^ and H_2_O_2_ act as signaling molecules, H_2_O_2_ is considered the main signaling compound because of its relative stability and sub-cellular localization.^34^ For example, H_2_O_2_ activates redox-signaling sensitive pathways such as MAPK and Rho kinase.^35^ H_2_O_2_ is tightly regulated by intracellular and extracellular enzymes, including CAT, which converts H_2_O_2_ into water and O_2_. Our findings showed that acute ethanol intake did not alter either H_2_O_2_ levels or CAT activity in the rat MAB.

Endothelial dysfunction is caused by an increase in ROS generation and a reduction of endothelial NO bioavailability, by increasing the oxidative inactivation of NO and/or by decreasing its synthesis. In the vascular endothelium NO is synthesized by eNOS. Akt, a serine/threonine kinase, phosphorylates eNOS thereby activating the enzyme.^36^ Phosphorylation of eNOS at Ser^1177^ residue is a critical requirement for eNOS activation, whereas phosphorylation at Thr^495^ residue leads to inactivation of the enzyme.^36^ Here we demonstrated that ethanol had no effect on the Akt/eNOS phosphorylation, which corroborated the observation that ethanol intake did not alter the concentration of nitrate in the rat MAB. Ethanol induces a transient increase in the activity of both SOD and CAT and this response can shift the balance ROS/NO towards NO levels.^37^ In this regard , ethanol-induced increase in antioxidant defenses could explain the lack of effect of ethanol on nitrate levels in the rat MAB. However, acute ethanol intake did not alter GSH levels or SOD and CAT activities in the rat MAB, indicating that the treatment did not affect the cellular antioxidant capacity.

NAD(P)H oxidase-derived ROS in the vasculature activate redox-sensitive targets such as RhoA/Rho kinase. Superoxide anion and H_2_O_2_ activate the RhoA/Rho kinase pathway, which represents an important class of redox-regulated signaling molecules in the cardiovascular system.^13^ The RhoA/Rho kinase pathway regulates many intracellular signaling pathways in the vasculature. Rho cycles between the GDP-bound inactive form located in the cytoplasm and the GTP-bound active form in the cell membrane,^38^ and RhoA translocation to the membrane is associated with its activation. The present findings showed that ethanol intake increased RhoA translocation, further suggesting an activation of the RhoA/Rho kinase pathway. Also , the fact that vitamin C prevented ethanol-induced RhoA translocation suggests that this response is mediated by ROS. This result is in agreement with previous findings showing that ROS, most notably O_2_^-^ and H_2_O_2_, are linked to the activation of the RhoA/Rho kinase pathway.^39^ To the best of our knowledge, this is the first study demonstrating a direct interaction among ethanol intake, NAD(P)H oxidase-derived ROS and the activation of the RhoA/Rho kinase signaling pathway. RhoA is abundantly expressed in VSMC and participates in vasoconstriction via phosphorylation of myosin light chain and sensitization of contractile proteins to calcium. Additionally, increased RhoA activation has been linked to endothelial dysfunction, increased peripheral vascular resistance and hypertension.^40^ Despite the activation of the RhoA/Rho kinase signaling pathway, acute ethanol intake did not affect the contractile response induced by phenylephrine or the endothelium-dependent relaxation induced by acetylcholine in the mesenteric artery. These results suggest that RhoA activation here described probably occurs before the onset of severe functional abnormalities.

Some limitations for the present study should be considered. In our study, all parameters were evaluated when ethanol reached its maximal plasma concentration. The period of action of ethanol in the vasculature after a single dose was not evaluated. Thus, studies on the time-course effect of a single dose administration of ethanol are of interest. Another point that should be considered is that while "binge drinking" in humans is broadly defined as the consumption of a large amount of ethanol (4-5 standard drinks) in a two-hour period, in our study the total amount of ethanol (1g/kg) was administered in a single dose.

Activation of NAD(P)H oxidase with subsequent increase in ROS generation and activation of redox-sensitive signalling pathways, such as the RhoA/Rho kinase pathway, are important events linked to vascular dysfunction and are described to play a role in the pathophysiology of several cardiovascular diseases.^9,11,13^ Binge drinking is associated with a heightened risk of cardiovascular events, such as stroke, sudden death, myocardial infarction, increased mortality after myocardial infarction,^2-4^ and with progression of carotid atherosclerosis.^1^ Importantly, changes in vascular biology are key mechanisms underlying the increased risk of adverse cardiovascular events induced by binge drinking.^6^ Thus, our findings raise the possibility that not only chronic ethanol intake is a risk factor for cardiovascular events, but also acute ethanol intake may increase the risk for vascular injury by increasing ROS generation and activation of redox-sensitive pathways. Altogether, these responses trigged by ethanol could predispose to the development of cardiovascular diseases.

## Conclusions

In summary, the major new finding of the present study is that acute ethanol intake induces activation of the RhoA/Rho kinase pathway by a mechanism that involves ROS generation. Additionally, we first demonstrated that ethanol activates NAD(P)H oxidase by induction of p47phox translocation and by redox-sensitive mechanisms in resistance arteries.
